# A remarkable new *Helophorus* species (Coleoptera, Helophoridae) from the Tibetan Plateau (China, Sichuan)

**DOI:** 10.3897/zookeys.718.21361

**Published:** 2017-12-04

**Authors:** Robert B. Angus

**Affiliations:** 1 Department of Life Sciences (Insects), The Natural History Museum, Cromwell Road, London SW7 5 BD, UK

**Keywords:** *Helophorus*, Helophoridae, new species, *Helophorus* s. str., *Meghelophorus*, *H. dracomontanus* sp. n., *H. jaechi* Angus, *H. kozlovi* Zaitsev, Tibetan Plateau, China, Sichuan

## Abstract

*Helophorus dracomontanus* **sp. n.** is described from the Tibetan Plateau near Kangding, Sichuan, China. It is a member of the subgenus Helophorus s. str. but the anterolateral part of the pronotum resembles subgenus Gephelophorus Sharp, 1915. The short metallic-black maxillary palpi with almost symmetrical apical segments are suggestive of subgenus Kyphohelophorus Kuwert, 1886, but the elytral flanks are narrower than the epipleurs, excluding the species from that subgenus. An adjusted key to *Helophorus* s. str. is given to identify the new species, as well as *H. jaechi* Angus, 1995 and *H. kozlovi* Zaitsev, 1908.

## Introduction

In the course of a beetle-collecting trip to the Kangding area of Sichuan in June-July 2016, two females of an unknown *Helophorus*, apparently a robust member of the *H. glacialis* Villa & Villa, 1833 species group, were taken. Despite further searching, no male was encountered. Study of the material back in the laboratory showed that the elytra had scutellary striae, and this, in conjunction with the fairly narrow elytral flanks, indicated that it was a member of the subgenus Helophorus s. str. It is described here as it is sufficiently distinctive to be easily recognised from females alone.

## Taxonomy

### Helophorus (Helophorus s. str.) dracomontanus sp. n.

Taxon classificationAnimaliaColeopteraHelophoridae

http://zoobank.org/87F18E76-D613-430B-A925-AA33A04EE4B6

#### Differential diagnosis.

Placed in the subgenus Helophorus s. str. because of its scutellary striae on the elytra, and elytral flanks clearly narrower than the epipleurs opposite the metaventrite. Distinguished from all known species of *Helophorus* s. str. by the short metallic black maxillary palpi with their apical segments almost symmetrical. The other known species of *Helophorus* s. str. with dark maxillary palpi with almost symmetrical apical segments (*H. (H. s. str.) niger* J. Sahlberg, 1880 and *H. (H. s. str.) khnzoriani* Angus, 1970) have the palpi longer and their pronota totally devoid of granules.

#### Description.

**Figure 1.**
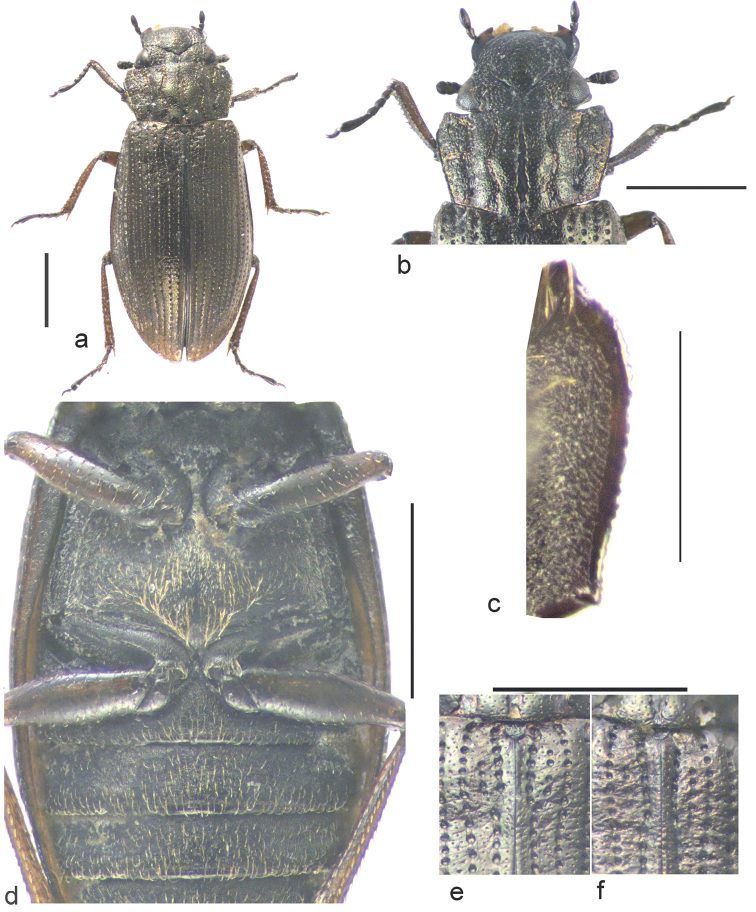
*Helophorus dracomontanus* sp. n. **a** holotype, dorsal **b** holotype head and pronotum, dorsal **c** paratype, lateral part of pronotum, ventral **d** paratype elytral epipleurs and flanks, metaventrite and abdomen, ventral **e, f** base of elytra and pronotum (dorsal) of holotype (**e**) and paratype (**f**) showing the scutellary striae. Scale bar: 1 mm (**a, b, d, e, f**); 0.5 mm (**c**).

General appearance: Fig. 1a; Head and pronotum: Fig. 1b; Underside of lateral area of pronotum: Fig. 1c. Ventral view of elytra, metaventrite and abdomen: Fig. 1d; Basal part of elytra: Fig. 1e, f.

Length: 4.3–4.4 mm; breadth: 1.9 mm. Metallic black, elytra with dark brown undertone and legs dark brown.

Head: strongly granulate, slightly shining with dark green-bronze reflection. Stem of Y-groove narrow linear, 2× wider than arms. Maxillary palpi metallic black with dark brown undertone, short, apical segment almost symmetrical oval. Antennae 9-segmented, brownish black, clubs darker.

Pronotum: widest about a fifth of the way from the anterior margin, abruptly narrowed anteriorly with the sides sinuate just behind anterior angles, but almost straight behind widest point, convergent to hind angles. Moderately arched, middle portion of internal intervals somewhat bulging, outer part of middle intervals and inner part of external intervals somewhat depressed. Granulation somewhat reduced on middle part of internal intervals, stronger on middle intervals and coarse on externals. Grooves shallow, mid groove over most of its length as wide as the diameter of 3 punctures, tapered to a point anteriorly, slightly constricted but parallel-sided in basal quarter. Floor of groove shining with a few small sparse punctures. Submedian grooves about half width of mid groove, angled outwards medially, reflexed a quarter of the way from each end. Floors shining, rugulose. Submarginals about as wide as mid groove, their sides irregular, floors rugose, shining. Marginal grooves narrow, effaced in anterior quarter. Narrow raised margins finely crenulate. Underside of pronotum with shining suprapleural area narrow at front, widest in anterior quarter, then evenly narrowed to hind angles.

Elytra: widest just behind middle, then tapered to rounded apex. Striae weakly impressed – strial punctures strong but not connected by grooves. Interstices flat with one or two rows of fine punctures, but interstices 1–3 rugose over basal quarter. Scutellary striae of 3–4 punctures, partly concealed by the rugosity. Surface of elytra with a V-shaped depression over basal quarter. Interstice 11 strongly keeled, flanks opposite the metaventrite about three quarters the width of the epipleurs.

Abdomen: ventrites black with erect pubescence. Apical margin of sternite 7 not denticulate.

Legs: dark brown, tarsi blackened apically. Rather short, tarsal hairs indistinct.

#### Holotype.

**Figure 2.**
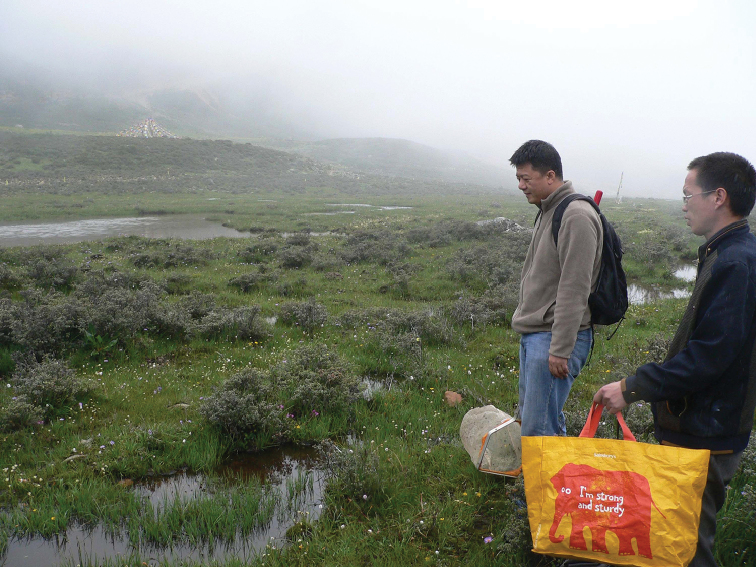
Habitat of *Helophorus dracomontanus* sp. n., Sichuan. Kangding County. Yalashenshan 30°12'17.22"N, 101°45'17.82"E. Small pools 4074 m above sea level. On the right is the driver and beside him Zhi-qiang Li. (For an account of this trip, see Angus 2017).

♀, CHINA, Sichuan. Kangding County. Yalashenshan 30°12'17.22"N 101°45'17.82"E. Small pools 4074 m a.s.l. (Fig. 2), R. B. Angus, F.-L. Jia & K. Chen. 27.vi.2016. In the Museum of Zoology, Sun Yat-sen University, Guangzhou, China (SYSU).

#### Paratype.

♀, data as holotype. In the Natural History Museum, London (BMNH).

#### Derivation of the name.

*dracomontanus* – Latin, mountain dragon. The species is named after Dr Fenglong Jia. The first part of his name, Feng – phoenix, is a Chinese homophone of Feng – mountain peak. The second part, Long, is straightforward – dragon.

## Discussion

*Helophorus dracomontanus* is placed in subgenus Helophorus s. str. because of its scutellary striae and elytral flanks not wider than the epipleurs. The suprapleural areas of the pronotum (pronotal epipleurs) are widest in basal quarter, distinctly narrower in front because of the sinuation of the lateral margins (Fig. 1c), and evenly narrowed to the hind angles. In this respect *H. dracomontanus* resembles the species of *Gephelophorus* Sharp, 1915. But in both of these (*H. sibiricus* Motschulsky, 1860 and *H. auriculatus* Sharp, 1884) the elytral flanks are at least as wide as the epipleurs. The metallic black maxillary palpi with almost symmetrical oval apical segments are shared by *H. (H. s. str.) niger* J. Sahlberg, 1880 and *H. (H. s. str.) khnzoriani* Angus, 1970, though the palpi are longer in these species, both of which have the pronota devoid of granulation. Neither of these species has the lateral margins of the pronotum sinuate behind the anterior angles. Its general appearance resembles a non-tuberculate H. (Kyphohelophorus) tuberculatus Gyllenhal, 1808 but in *Kyphohelophorus* the elytral flanks are much wider than the epipleurs.

Shining, often metallic, black maxillary palpi are fairly unusual in *Helophorus*. *H. niger* is associated with very dark substratum (e.g. Sahlberg 1880) and *H. khnzoriani* is a high-altitude species (Angus 1970), occurring in areas which are frequently dark and unvegetated after snow-melt, and this is also true of the *H. glacialis* Villa & Villa, 1833 and other species of the *Helophorus glacialis* and *H. guttulus* Motschulsky, 1860 species groups of the former subgenus Atractohelophorus Kuwert, 1886 (Angus et al. 2017). Finally, *H. tuberculatus* is typically found in recently burned (and thus) black areas and is a superb charcoal-mimic (Angus 1992). It therefore seems that these dark metallic palpi are associated with adaptation to dark ground, generally at high altitude or latitude.

In the key to the world species of Helophorus s. str. (Meghelophorus Kuwert, 1886) (Angus 1970), and also in the key to the Western Palaearctic species (Angus 1992), *H. dracomontanus* would run to couplet 8 because abdominal sternite 7 lacks square-ended teeth and the head has distinct granules over most of its surface. *H. dracomontanus*, as well as *H. kozlovi* Zaitsev, 1908 (not available when the 1970 key was written) and *H. jaechi* Angus, 1995 can be accommodated by substitution of a new Couplet 8 followed by three additional ones, 8’ – 8”’, as follows:

**Table:** 

8	Maxillary palpi short, metallic black, apical segment almost symmetrical oval	***dracomontanus* sp. n.**
–	Maxillary palpi longer, at least in part brownish or yellow, apical segment clearly asymmetrical	**8**’
8’	Internal intervals of pronotum without granules. Elytra brown with extensive darker mottling as well as dark marks representing the sutural V-mark. Aedeagus either similar to that of *H. aequalis* (Angus, 1995, fig. 14) or with the outer margins of the parameres more curved	***kozlovi* Zaitsev, 1908**
–	Internal intervals of the pronotum extensively granulate	**8**”
8”	Conspicuously elongate beetles with relatively small pronota (Angus, 1995, figs 1, 5). Aedeagus with parameres narrow, slightly incurved apically (Angus, 1995, fig. 13)	***jaechi* Angus, 1995**
–	Less elongate beetles, pronotum relatively slightly larger (Angus, 1992, fig. 26 a, b)	**8**’’’ (*H. bergrothi* J. Sahlberg, 1880, *H. strandi* Angus, 1970, *H. hammondi* Angus, 1980), as from couplet 8 in the published keys

## Supplementary Material

XML Treatment for Helophorus (Helophorus s. str.) dracomontanus
